# *Aliidiomarina halalkaliphila* sp. nov., a haloalkaliphilic bacterium isolated from a soda lake in Inner Mongolia Autonomous Region, China

**DOI:** 10.1099/ijsem.0.005263

**Published:** 2022-03-04

**Authors:** Ming Yang, Qiong Xue, Zhenqiang Zuo, Jian Zhou, Shengjie Zhang, Ming Li, Heng Zhou, Manqi Zhang, Sumit Kumar, Wei Li, Guiying Chen, Dahe Zhao, Hua Xiang

**Affiliations:** ^1^​ State Key Laboratory of Microbial Resources, Institute of Microbiology, Chinese Academy of Sciences, 100101, Beijing, PR China; ^2^​ Sichuan Normal University, Sichuan 610101, PR China; ^3^​ University of Chinese Academy of Sciences, Beijing 100049, PR China; ^4^​ Enzyme and Microbial Biochemistry Lab, Department of Chemistry, Indian Institute of Technology, Delhi, India

**Keywords:** *Aliidiomarina*, soda lake, haloalkaliphilic bacterium, polyphasic taxonomy, whole-genome sequence

## Abstract

A haloalkaliphilic strain (IM 1326^T^) was isolated from brine sampled at a soda lake in the Inner Mongolia Autonomous Region, China. Cells of the strain were rod-shaped and motile. Strain IM 1326^T^ was able to grow at 4–42 °C (optimum, 37 °C) with 0–13.0 % (w/v) NaCl concentrations (optimum at 4.0–6.0 %) and at pH 7.5–11.0 (optimum at 9.0–10.0). The 16S rRNA gene phylogenetic analysis revealed that the isolate belongs to the genus *Aliidiomarina* and is closely related to the type strains of *Aliidiomarina sanyensis* (95.8 % sequence similarity), *Aliidiomarina shirensis* (95.7 %), *Aliidiomarina iranensis* (95.4 %) and *Aliidiomarina haloalkalitolerans* (95.3 %). The whole genome of strain IM 1326^T^ was sequenced, and the genomic DNA G+C content was 49.7 mol%. Average nucleotide identity, average amino acid identity and digital DNA–DNA hybridization values between the isolate and the related *Aliidiomarina* species were 68.1–84.9 %, 76–78 % and 18.4–20.4 %, respectively. The respiratory quinone was ubiquinone-8. The polar lipid profile included diphosphatidylglycerol, phosphatidylglycerol, phosphatidylethanolamine and one unidentified aminophospholipid. The predominant cellular fatty acids were summed feature 9 (10-methyl-C_16 : 0_/iso-C_17 : 1_ *ω*9*c*, 22.2 %), iso-C_15 : 0_ (16.1 %) and iso-C_17 : 0_ (13.1 %). Based on the results of phylogenetic analysis, genome relatedness, and the physiological and chemotaxonomic properties of the isolate, strain IM 1326^T^ is considered to represent a novel species of the genus *Aliidiomarina *, for which the name *Aliidiomarina halalkaliphila* sp. nov. is proposed (type strain IM 1326^T^=CGMCC 1.17056^T^=JCM 34227^T^).

## Introduction

The genus *Aliidiomarina* belongs to the family *Idiomarinaceae *, order *Alteromonadales,* class *Gammaproteobacteria* [1]. This genus has two sister genera named *Idiomarina* and *Pseudidiomarina* in the List of Prokaryotic names with Standing in Nomenclature. Despite the reclassification of the genus *Pseudidiomarina* into the genus *Idiomarina *, the species of both genera could not be distinguished from each other using phenotypic or chemotaxonomic characteristics [2], and recently the genus *Pseudidiomarina* was reinstated following the results of genome-based analysis [3]. The genus *Aliidiomarina* was close to the genus *Pseudidiomarina* in the phylogenetic tree based on the 16S rRNA genes. However, it is distant from both sister genera in the phylogenomic tree based on the 78 core genes [3]. At the time of writing, a total of ten species of this genus were validly published, including *Aliidiomarina taiwanensis* [1], *Aliidiomarina haloalkalitolerans* [4], *Aliidiomarina shirensis* [5], *Aliidiomarina minuta* [6], *Aliidiomarina iranensis* [7], *Aliidiomarina sanyensis* [8], *Aliidiomarina soli* [9], *Aliidiomarina maris* [10], *Aliidiomarina celeris* [11] and *Aliidiomarina sedimenti* [12]. These species were isolated from a wide variety of habitats, including seawater [1, 4], saline lakes [5, 6], coastal-marine wetland [7], *Spirulina platensis* cultivation pool [8], soil [9] and sediments [10–12]. Members of the genus *Aliidiomarina* are Gram-stain-negative, rod-shaped, aerobic and require sodium ions for growth. The major isoprenoid quinone is ubiquinone-8, and iso-branched fatty acids including iso-C_17  :  0_, summed feature 9 (comprising iso-C_17  :  1_ *ω*9*c* and/or 10-methyl C_16  :  0_) and iso-C_15  :  0_ are the major cellular fatty acid components [1]. This article describes a haloalkaliphilic strain isolated from a soda lake. The strain is proposed as a novel species of the genus *Aliidiomarina *.

## Isolation and maintenance

Strain IM 1326^T^ was isolated from a brine sample of a soda lake (Hutong Qagan Lake) from the Ordos, Inner Mongolia Autonomous Region of China, in July 2017. The sampling site was located at the position of 39° 12′ 14″ N, 109° 0′ 8″ E, and an altitude of 1250 m. The total salinity of the lake water was 26%, while the pH was 9.95. The brine sample was stored in a sterilized plastic bottle and transported to the laboratory at ambient temperature. The sample was incubated at 37 °C for 2 weeks after being inoculated in LN liquid medium (originally designed for microbe using light by refering neutral oligotrophic medium) composed of (per litre): 15.0 g NaCl, 0.5 g yeast extract (Difco), 6.0 g NaHCO_3_, 4.0 g Na_2_CO_3_, 0.2 g NH_4_Cl, 2.0 g KCl, 0.25 g fish peptone, 0.38 g sodium formate, 0.25 g sodium acetate, 0.25 g sodium pyruvate, 1.0 g MgSO_4_·7H_2_O, 0.08 g CaCl_2_, 56 mg FeSO_4_·7H_2_O, and pH was adjusted to 9.5 with 1 M NaOH. The enrichment culture was spread on agar plates of the same medium (15.0 g agar per litre) and incubated at 37 °C for 5 days. Pure cultures were obtained by repeated subculturing of a single colony on the same solid medium. A circular, convex and light brown colony was obtained on LN plates after 5 days of incubation at 37 °C. The strain was routinely cultured at 37 °C in LN medium. Stock culture of the isolate was preserved in LN medium with 20 % glycerol (v/v) at −80 °C.

## Phylogenetic analysis based on 16S rRNA gene sequences

The phylogenetic position of strain IM 1326^T^ was determined through 16S rRNA gene sequence analysis. The 16S rRNA gene sequence was amplified using oligonucleotide primers complementary to highly conserved regions of bacterial 16S rRNA genes. The forward and reverse primers were 27F (5′-AGAGTTTGATCCTGGCTCAG-3′) and 1492R (5′-GGTTACCTTGTTACGACTT-3′), respectively [13]. The amplified product was cloned in the pMD-18T vector (Takara), and the recombinant plasmids were transformed into *Escherichia coli* JM109 (BioMed) cells. Sanger sequencing was performed by Witon Information Technology Co., Ltd. (Beijing, PR China). Sequences were preliminarily analysed by the blast program (https://blast.ncbi.nlm.nih.gov/Blast.cgi) and the EzBioCloud webserver (www.ezbiocloud.net), and the closely related sequences were selected as references from the list of hits with valid names. The maximum-likelihood, neighbour-joining, minimum-evolution and maximum-parsimony methods were used to reconstruct the phylogenetic trees in mega 6.0 software [14]. The robustness of the phylogenetic tree topology was evaluated by bootstrap analyses based on 1000 replicates [15].

**Fig. 1.**
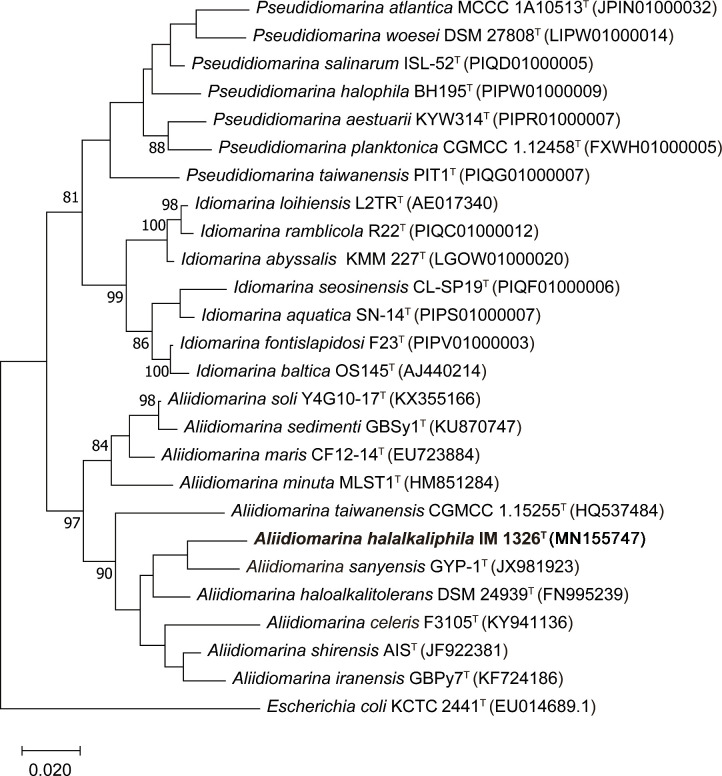
Maximum-likelihood phylogenetic tree based on 16S rRNA gene sequences showing the relationship between strain IM 1326^T^ and related taxa. Bootstrap values (based on 1000 replicates) of more than 70 % are shown at the branching point. The sequence of *Escherichia coli* KCTC 2441^T^ was used as an outgroup. GenBank accession numbers are given in parentheses. Bar, 0.02 substitutions per nucleotide position.

This study determined the nearly full-length 16S rRNA gene sequence (1503 bp) of strain IM 1326^T^. The similarities between strain IM 1326^T^ and the most closely related species *A *. *sanyensis *, *A. shirensis *, *A. iranensis *, *A *. *haloalkalitolerans *, *A *. *celeris *, *A. minuta* and *A *. *taiwanensis* (the type species of this genus) were 95.8, 95.7, 95.4, 95.3, 95.0, 94.7 and 93.6%, respectively. In the maximum-likelihood tree, strain IM 1326^T^ fell within a clade comprising species of the genus *Aliidiomarina *. The subclade was composed of *A *. *taiwanensis* AIT1^T^, *A. sanyensis* GYP-17^T^, and *A *. *haloalkalitolerans* AK5^T^ (Fig. 1). Considering the low bootstrap values (not shown if less than 70%), the topology of the phylogenetic tree was not stable. The topology and phylogenetic relationship were also supported by the neighbour-joining, minimum-evolution and maximum-parsimony phylogenetic trees (Figs S1–S3, available in the online version of this article). In addition, the genera *Pseudidiomarina* and *Idiomarina* were closer in most phylogenetic trees (Figs 1, S1 and S2), apart from the maximum-parsimony tree (Fig. S3). Moreover, the bootstrap values were lower than 70 % between the genus *Aliidiomarina* and the clade of the genera *Pseudidiomarina* and *Idiomarina *. This finding is different from the previous report using FastTree with JTT+CAT parameters [3]. Altogether, strain IM 1326^T^ was likely to represent a novel species of the genus *Aliidiomarina *.

## Genome features and phylogenomic analysis

The draft genome of strain IM 1326^T^ was sequenced by Biomarker Technologies (Beijing, PR China) for genome-level phylogenetic analysis. Briefly, the genomic DNA was extracted using the cetyltrimethylammonium bromide (CTAB) method, and purity was ascertained using a Qubit 3.0 fluorometer (Invitrogen). After the construction of the DNA library, the genome was sequenced using an Illumina NovaSeq platform. Genome assembly was carried out using SPAdes version 3.13.0. The draft genome sequence has been deposited with accession number VJWL00000000 in the GenBank of the NCBI nucleotide database (www.ncbi.nlm.nih.gov/genome) and annotated using the NCBI Prokaryotic Genome Annotation Pipeline version 4.7 (www.ncbi.nlm.nih.gov/genome/annotation_prok/). DNA G+C content and other detailed information were obtained from the genome sequence. The average nucleotide identity (ANI) percentages with other species of the genus *Aliidiomarina* were calculated using the blast (ANIb) and MUMmer (ANIm) algorithms from the JSpecies Web Server (http://jspecies.ribohost.com/jspeciesws) [16]. The average amino acid identity (AAI) values were calculated using the online tool ANI/AAI-Matrix (http://enve-omics.ce.gatech.edu/g-matrix/), a genome-based distance matrix calculator. In addition, genome-to-genome distance analysis via digital DNA–DNA hybridization (dDDH) was performed with the Genome-to-Genome Distance Calculator (http://ggdc.dsmz.de/distcalc2.php) using the method described by Meier-Kolthoff *et al*. [17]. Furthermore, a phylogenomic tree was reconstructed based on 120 bacterial conserved single-copy genes obtained by employing the Genome Taxonomy Database [18].

Genome sequencing of strain IM 1326^T^ resulted in raw data of 3.06 Gb with 1189× genome coverage. Sequence reads were *de novo* assembled into 19 contigs and 12 scaffolds. The genomic size was 2.57 Mb. The genomic DNA G+C content of strain IM 1326^T^ was estimated to be 49.7 mol%, which was similar to that of other species of the genus *Aliidiomarina* (46.3–52.1 mol%) and slightly higher than that of *A *. *taiwanensis* AIT1^T^ (48.7 mol%), the type species of the genus *Aliidiomarina* (Table 1). The detailed genomic information is presented in Table S1. The value of genome relatedness between strain IM 1326^T^ and its closely related species ranged from 68.1 to 72.6 % for ANIb, 80.7 to 84.9 % for ANIm, 76 to 78 % for AAI, and 16.2 to 22.7 % for dDDH (Table 2). The percentage similarity values with *A *. *sanyensis* GYP-17^T^, the most closely related species, were 72.6 % for ANIb, 83.5 % for ANIm, 78 % for AAI, and 18.4 % for dDDH. The obtained similarity percentage values are lower than the standard thresholds for species delineation, i.e., 95 % for ANI, 95 % for AAI and 70 % for dDDH.

**Table 1.** Differentiating characteristics of strain IM 1326^T^ and the ten type strains of the genus *Aliidiomarina
* Strains: 1, IM 1326^T^ (data from this study); 2, *A *. *taiwanensis* CGMCC 1.15255^T^; 3, *A *. *haloalkalitolerans* DSM 24939^T^; 4, *A *. *sanyensis* GYP-17^T^; 5, *A. shirensis* AIS^T^; 6, *A. minuta* MLST1^T^; 7, *A. iranensis* GBPy7^T^; 8, *A. soli* Y4G10-17^T^; 9, *A. maris* CF12-14^T^; 10, *A. celeris* F3105^T^; 11, *A. sedimenti* GBSy1^T^. +, Positive; −, negative; w, weakly positive; nd, not detected; DPG, diphosphatidylglycerol; PG, phosphatidylglycerol; PE: phosphatidylethanolamine; APL, unidentified aminophospholipid; PL, unidentified phospholipid. DNA G+C content of strain IM 1326^T^ was obtained from the genomic sequence. Data for *A *. *taiwanensis* CGMCC 1.15255^T^, and *A *. *haloalkalitolerans* DSM 24939^T^ were taken from Srinivas *et al*. [2], and some experiments were repeated under identical conditions (marked by *). Data for other species were taken from the literature: *A. sanyensis* GYP-17^T^ [6], *A. shirensis* AIS^T^ [3], *A. minuta* MLST1^T^ [4], *A. iranensis* GBPy7^T^ [5], *A. soli* Y4G10-17^T^ [7], *A *. *maris* CF12-14^T^ [8], *A *. *celeris* F3105^T^ [9], and *A. sedimenti* GBSy1^T^ [10].

Characteristics	1	2	3	4	5	6	7	8	9	10	11
Cell size (um)	0.3–0.6×0.8–2.1	0.4–0.86×1.5–3.0	0.8–1.06×1.0–1.5	0.4–0.56×0.7–2.0	0.8–1.76×0.4–0.5	0.4–1.0×1.0–3.0	0.6–0.96×1.8–2.2	0.1–0.3×1.2–2.4	0.3–0.6×0.8–2.4	0.6–0.9×1.5–2.6	0.3–0.4×1.2–1.7
Colony colour	Light brown	Light brown*	Creamish*	Dark brown	Pale yellow	Cream	Buttery	Yellow	White to light yellow	Brown	Non-pigmented
Salinity growth range (%)	0–13.0	0.5–10.0*	0.5–12.0*	1.0–10.0	0.5–15.0	0–15.0	1.0–15.0	1.0–14.0	1.0–15.0	1.0–8.0	1.0–15.0
Optimum salinity for growth (%)	4.0–6.0	1.5–5.0*	0.5–5.0*	3.0–7.0	1.0–10.0	7.0–10.0	3.0	1.0–5.0	2.0–3.0	2.0–3.0	5.0
Temperature range for growth (°C)	4–42	4–40*	10–50*	10–45	1–45	10–45	4–45	4–45	4–42	4–45	4–40
Optimum temperature for growth (°C)	37	30–40*	30–37*	30	30–35	30	37	30	30–35	37	34
pH range for growth	7.5–11.0	7.0–11.0*	7.0–11.0*	7.0–9.0	5.0–9.5	6.0–11.0	7.0–10.0	6.0–12.0	6.0–11.5	6.5–9.5	7.0–10.0
Optimum pH for growth	9.0–10.0	8.0*	8.0–10.0*	8.0	7.5–8.0	9.5	8.5	9.0	8.0–9.5	7.5	8.5
Indole production	−	+	−	−	−	nd	−	−	−	−	−
Utilization of carbon sources:											
d-Glucose	−	+*	w*	−	−	+	−	−	−	−	−
d-Sorbitol	−	+*	w*	−	−	−	−	−	−	−	−
d-Fructose	−	+*	w*	−	−	−	−	+	−	+	−
Lactose	−	+*	-*	−	−	−	−	−	−	−	−
Maltose	−	+*	w*	−	+	−	−	+	−	−	−
d-Mannose	−	+*	+*	+	−	−	−	−	−	−	−
d-Galactose	−	+*	-*	+	−	−	−	−	−	−	−
Glycerol	−	+*	+*	+	−	−	−	−	−	−	−
Ribose	−	+*	-*	−	−	−	−	−	−	−	−
Sucrose	−	+*	w*	−	+	−	−	+	−	−	−
Trehalose	−	+*	w*	−	+	−	−	−	−	−	−
l-Histidine	+	+*	-*	−	−	−	−	−	−	−	−
Hydrolysis of:											
Tween 80	−	−	+	+	−	+	−		−	−	+
Quinone composition	Q-8	Q-8, Q-9*	Q-8	Q-8	Q-8, Q-7	Q-8	Q-8	Q-8, Q-9	Q-8	Q-8	Q-8, Q-9, Q-10
Major polar lipids	DPG, PG, PE, APL	DPG, PE, PG, PL5, APL1*	DPG, PE, PG, PL1–PL4*	PE, PL1–PL3	DPG, PG, PE, PL	DPG, PG, PE	PG, DPG, PE, PL1, APL1	DPG, PG, PE	DPG, PG, PE	DPG, PG, PE, AL, APL1–2, L1–2	DPG, PG, PE, PL1–PL3
DNA G+C content (mol%)	49.7	48.7	49.3	50.8	46.3	48.7	46.8	49.6	50.1	51.6	52.1

*Determined in this study.

**Table 2.** Genome relatedness based on average nucleotide identity (ANI), average amino acid identity (AAI), and digital DNA–DNA hybridization (dDDH) values among strain IM 1326^T^ and species of the genus *Aliidiomarina *. Strain: 1, IM 1326^T^; 2, *A *. *taiwanensis* CGMCC 1.15255^T^; 3, *A *. *haloalkalitolerans* DSM 24939^T^; 4, *A *. *sanyensis* GYP-17^T^; 5, *A*. AIS^T^; 6, *A. minuta* MLST1^T^; 7, *A. iranensis* GBPy7^T^; 8, *A. soli* Y4G10-17^T^; 9, *A. maris* CF12-14^T^; 10, *A. celeris* F3105^T^; 11, *A. sedimenti* GBSy1^T^. ANIb and ANIm values were calculated by blast and MUMmer algorithms, respectively. AAI values were calculated by ANI/AAI-Matrix. dDDH values were obtained by calculating genome-to-genome distance.

ANIb	1	2	3	4	5	6	7	8	9	10	11
**1**	–										
**2**	68.2	–									
**3**	70.6	68.7	–								
**4**	72.6	68.2	70.5	–							
**5**	70.8	68.3	71.0	70.1	–						
**6**	68.1	67.3	68.3	67.7	68.3	–					
**7**	70.6	68.2	71.0	69.7	78.2	68.3	–				
**8**	68.1	67.6	68.5	68.0	68.1	70.0	68.1	–			
**9**	68.5	68.0	69.2	68.4	68.5	70.1	68.6	71.5	–		
**10**	69.0	69.9	69.7	68.8	69.3	67.9	69.4	68.1	69.0	–	
**11**	68.1	67.4	68.5	68.1	68.2	71.0	68.1	95.1	71.5	68.2	–

**ANIm**	**1**	**2**	**3**	**4**	**5**	**6**	**7**	**8**	**9**	**10**	**11**
**1**	–										
**2**	80.7	–									
**3**	84.9	84.2	–								
**4**	83.5	87.5	85.8	–							
**5**	84.5	85.7	84.0	85.4	–						
**6**	84.2	84.3	84.1	84.3	84.6	–					
**7**	83.9	85.0	84.1	84.9	84.6	84.8	–				
**8**	84.0	84.4	84.8	84.7	84.9	84.5	85.3	–			
**9**	84.3	84.7	86.4	87.5	85.3	84.7	85.7	85.5	–		
**10**	84.3	85.5	85.2	85.5	84.9	85.6	86.2	85.3	85.7	–	
**11**	84.0	84.3	85.0	84.6	84.9	84.6	84.7	95.2	84.5	84.9	–

**AAI**	**1**	**2**	**3**	**4**	**5**	**6**	**7**	**8**	**9**	**10**	**11**
**1**	–										
**2**	76	–									
**3**	78	77	–								
**4**	78	82	80	–							
**5**	78	79	77	79	–						
**6**	76	76	77	76	77	–					
**7**	78	77	77	78	81	77	–				
**8**	76	77	77	77	77	78	77	–			
**9**	76	77	78	80	77	78	78	79	–		
**10**	78	79	78	79	78	76	79	77	78	–	
**11**	76	77	77	76	77	77	77	95	78	76	–

**dDDH**	**1**	**2**	**3**	**4**	**5**	**6**	**7**	**8**	**9**	**10**	**11**
**1**	–										
**2**	18.6±2.3	–									
**3**	19.2±2.3	20.1±2.3	–								
**4**	18.4±2.2	20.7±2.3	19.9±2.3	–							
**5**	18.6±2.2	21.4±2.3	19.1±2.3	19.3±2.3	–						
**6**	19.2±2.3	18.4±2.2	19.5±2.3	18.6±2.3	19.2±2.2	–					
**7**	19.0±2.2	19.6±2.3	19.3±2.2	19.1±2.2	21.9±2.3	19.1±2.3	–				
**8**	18.6±2.2	19.8±2.3	20.3±2.3	20.4±2.3	20.5±2.3	18.1±2.2	20.1±2.3	–			
**9**	20.1±2.3	20.4±2.3	21.9±2.3	20.3±2.3	20.8±2.3	18.8±2.3	21.4±2.3	18.6±2.2	–		
**10**	20.4±2.3	18.5±2.2	20.6±2.3	20.5±2.3	20.5±2.3	19.1±2.3	21±2.3	19.2±2.3	21.1±2.3	–	
**11**	19.7±2.3	19.8±2.3	19.8±2.3	19.7±2.3	18.6±2.2	18.3±2.3	20.7±2.3	18.7±2.3	18.3±2.2	19.4±2.3	–

**Fig. 2.**
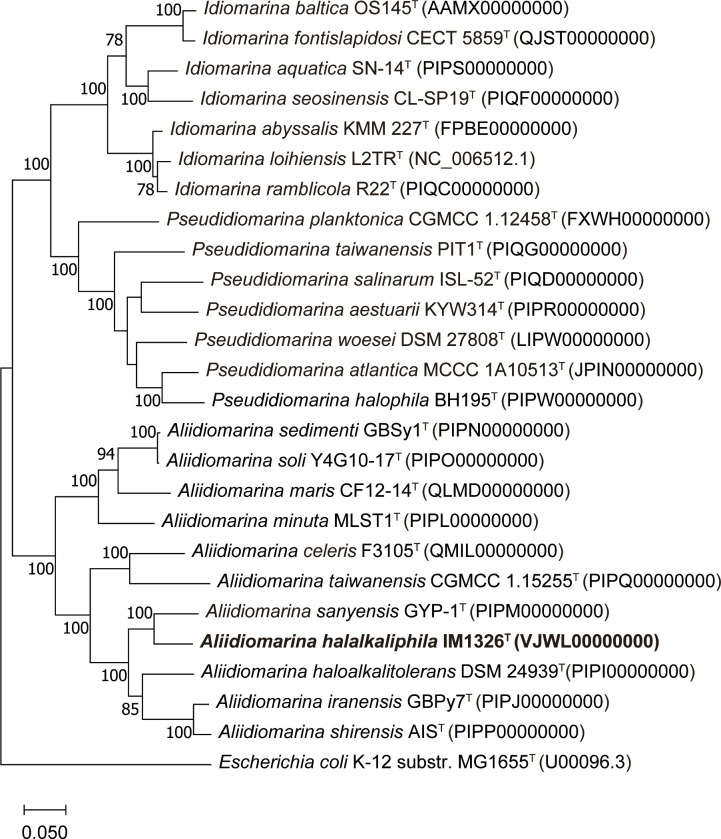
Maximum-likelihood phylogenetic tree based on 120 bacterial conserved single-copy gene sequences showing the relationship between strain IM 1326^T^ and related taxa. Bootstrap values (based on 1000 replicates) of more than 70 % are shown at the branching point. The sequence of *Escherichia coli* K-12 substr. MG1655^T^ was used as an outgroup. GenBank accession numbers are shown in parentheses. Bar, 0.05 substitutions per amino acid position.

The maximum-likelihood phylogenomic tree reconstruction based on 120 bacterial conserved single-copy genes revealed that strain IM 1326^T^ was located in the *Aliidiomarina* clade and separated from other species (Fig. 2). The bootstrap value between strain IM 1326^T^ and *A. sanyensis* GYP-17^T^ was 100%, suggesting it was a novel species of *Aliidiomarina *. The neighbour-joining, minimum-evolution and maximum-parsimony phylogenomic trees displayed similar phylogenetic positions (Figs S4–S6). In addition, the phylogenomic trees based on 120 bacterial conserved single-copy proteins agreed with the tree based on the 78 concatenated core protein sequences of the family *Idiomarinaceae* [3]. Therefore, the results confirmed that IM 1326^T^ represents a novel species in the genus *Aliidiomarina *.

The type species of the genus *Aliidiomarina *, *A *. *taiwanensis* CGMCC 1.15255^T^ (=AIT1^T^, obtained from the China General Microbiological Culture Collection Center), and *A *. *haloalkalitolerans* DSM 24939^T^ (=AK5^T^, obtained from the Leibniz Institute German Collection of Microorganisms and Cell Cultures) were selected as reference species to compare physiological characteristics and fatty acid compositions. *A *. *taiwanensis* CGMCC 1.15255^T^ and *A *. *haloalkalitolerans* DSM 24939^T^ were cultured using marine broth medium 2216E [peptone (5.0 g l^−1^), yeast extract (1.0 g l^−1^), ferric citrate (0.1 g l^−1^), NaCl (19.45 g l^−1^), MgCl_2_ (5.98 g l^−1^), Na_2_SO_4_ (3.24 g l^−1^), CaCl_2_ (1.8 g l^−1^), KCl (0.55 g l^−1^), Na_2_CO_3_ (0.16 g l^−1^), KBr (0.08 g l^−1^), SrCl (34.0 mg l^−1^), boric acid (22.0 mg l^−1^), sodium silicate (4.0 mg l^−1^), sodium fluoride (2.4 mg l^−1^), NaNO_3_ (1.6 mg l^−1^), Na_2_HPO_4_ (8.0 mg l^−1^), pH 7.4–7.8] at 30 °C.

## Physiological and chemotaxonomic characteristics

The physiological and chemotaxonomic characteristics of strain IM 1326^T^ were examined by using routine cultivation methods on HM medium containing (g l^−1^): NaCl (30.0), KCl (2.0), MgSO_4_·7H_2_O (1.0), NaBr (0.23), CaCl_2_ (0.27), NaHCO_3_ (0.06), FeCl_3_ (trace), tryptone (5.0), yeast extract (10.0) and agar (15.0 for solid medium) [19] at 37 °C and pH adjusted to pH 8.5 before sterilization. The Gram staining analysis was performed according to the method described by Dussault [20]. Morphology and cell size of the cells were observed under a scanning electron microscope (Hitachi H-700A). Motility was observed using an Olympus BX 51 microscope equipped with phase-contrast optics. The flagellum was observed under a transmission electron microscope. The temperature range for growth was determined at 1, 4, 15, 20, 25, 30, 34, 37, 40, 42, 45 and 50 °C in HM medium containing 3 % (w/v) NaCl at pH 9.0. The salinity range for growth was observed with 0–15.0 % (w/v) NaCl at intervals of 1.0 % (w/v). After the identification of the optimum salinity, NaCl concentration was changed to 4 % (w/v). The pH range for growth was determined from pH 5.0 to 12.0 at intervals of 0.5 pH units using the following buffers (25 mM concentration): MES (for pH 5.0–6.0), PIPES (pH 6.5–7.0), HEPES (pH 6.8–8.0), Tricine (pH 7.4–8.8), CHES (pH 8.6–10.0) and CAPS (pH 9.7–12.0). Anaerobic growth was tested in the presence of 5.0 % (w/v) nitrate, 3.0 % (w/v) l-arginine and 5.0 % (v/v) DMSO in stoppered tubes. Tests for catalase and oxidase activities and the hydrolysis of starch, gelatin, casein, and Tweens 20, 40, 60, and 80 were carried out using routine cultivation methods on HM medium with 4 % (w/v) NaCl as described by Harrigan *et al*. [21, 22]. H_2_S formation was detected using a filter-paper strip impregnated with lead acetate [23]. Indole production from tryptophan, the utilization of sugars and organic acids, and the Voges–Proskauer test were assessed as described by Oren *et al*. [24]. In brief, the utilization of sole carbon and energy sources was detected using minimal medium (tryptone was omitted and yeast extract was decreased to 1 g l^−1^) with various supplementary carbon sources and then comparing the cell density. Acid production from sugars and alcohols was determined, and the substrates used were d-glucose, d-galactose, sucrose, d-sorbitol, mannose, maltose, starch, d-fructose, l-sorbose, ribose, trehalose, d-xylose and glycerol. Production of acid from the above-mentioned carbohydrates and sugar alcohols was tested on the growth medium supplemented with 10.0 g l^−1^ of the substrate. The pH of the cultures was measured with a glass electrode at the end of the incubation period and compared with the initial pH and the pH of the control tube without substrate. Reduction of nitrate and nitrite were detected by using sulfanilic acid and α-naphthylamine reagent [25]. All tests were performed in triplicate along with the reference species under identical conditions.

Strain IM 1326^T^ was Gram-stain-negative. The cells were approximately 0.3–0.6 µm wide and 0.8–2.1 µm long (Fig. S7). The isolate can move, but no flagellum was observed under the transmission electron microscope (Fig. S8). Furthermore, the type of motility was explored. There were no rafts of cells and isolated cells were observed in the microscope image of the edge of colonies (Fig. S9), indicating that the cells did not move by gliding; therefore, further exploration is necessary. The colonies were light brown when grown on HM medium (pH 8.5) for 24 h at 37 °C. Strain IM 1326^T^ was able to grow in presence of 0–13.0 % NaCl concentration (optimum, 4.0–6.0 %), at 4–42 °C (optimum, 37 °C) and pH 7.5–11.0 (optimum, pH 9.0–10.0). Moreover, it showed more tolerance to alkalinity (up to pH 11.0) than most of the reference species. Strain IM 1326^T^ could hydrolyse gelatin, casein and different kinds of Tweens, e.g. Tween 20, 40 and 60, but could not hydrolyse starch. In addition, strain IM 1326^T^ was positive for nitrate reduction, but negative for nitrite reduction. Cells of strain IM 1326^T^ were oxidase- and catalase-positive. Correspondingly, a respiratory nitrate reductase operon (including four genes FM042_RS01865 to FM042_RS01880), NarK family nitrate/nitrite transporter (FM042_RS01935) and catalase (FM042_RS03320 and FM042_RS09450) were annotated in the genome. However, the draft genome did not contain the gene for amylase and nitrite reduction. Strain IM 1326^T^ could produce H_2_S from cysteine but did not produce indole from tryptophan. The detailed morphological, physiological and biochemical characteristics of strain IM 1326^T^ are provided in the species description and Table 1.

Cells grown on HM plates for 2 days at 37 °C were harvested for the cellular fatty acid analysis. The fatty acids were extracted according to the standard midi protocol (Sherlock Microbial Identification System, version 6.0). The cellular fatty acid profiles of strain IM 1326^T^, *A *. *taiwanensis* CGMCC 1.15255^T^ and *A *. *haloalkalitolerans* DSM 24939^T^ are shown in Table S2. The predominant fatty acids detected in strain IM 1326^T^ were 10-methyl-C_16 : 0_/iso-C_17 : 1_ *ω*9*c* (summed feature 9, 22.2 %), iso-C_15 : 0_ (16.1 %) and iso-C_17 : 0_ (13.1 %). These are also the dominant fatty acids in the two reference strains. In addition, we observed that, contrary to the original report, the content of C_16 : 0_ was high (up to 13.2 %) in *A *. *taiwanensis* CGMCC 1.15255^T^ [1]. However, the amount of C_16 : 0_ was lower in IM 1326^T^ and *A *. *haloalkalitolerans* DSM24939^T^ (Table S2). These data on the fatty acids composition of two reference species were similar to the previous report [2]. Despite the divergence in results from the different labs, all three species contained the major fatty acids of the genus *Aliidiomarina* [1].

Polar lipids of strain IM 1326^T^ were extracted by a phenol–chloroform system and analysed by using two-dimensional thin-layer chromatography (TLC) as described previously [26]. Merck silica gel 60 F254 aluminum-backed thin-layer plates were used for the TLC analysis. The first and second dimensional TLC solvents were chloroform–methanol–water (65 : 25 : 4, by vol.) and chloroform–methanol–acetate–water (80 : 12 : 15 : 4, by vol.), respectively. The reagent sulfuric acid–ethanol (1 : 1, v/v) was used to detect total polar lipids. According to the TLC chromatogram, the major polar lipids of strain IM 1326^T^ were diphosphatidylglycerol, phosphatidylglycerol, phosphatidylethanolamine and one unidentified aminophospholipid (Fig. S10). The polar lipid composition of two reference species (including the type species) were identified under identical experimental condition (Fig. S10). Most of the *Aliidiomarina* species contained diphosphatidylglycerol, phosphatidylglycerol and phosphatidylethanolamine (Table 1). The above findings are in unison with the polar lipid compositions of the genus *Aliidiomarina* [1].

Respiratory quinones were extracted from lyophilized cells, and the extracts were purified by TLC and analysed by high-performance liquid chromatography according to the method of Collins [27]. Strain IM 1326^T^ contained ubiquinone-8 as the sole respiratory quinone (Table 1). However, a minor quinone of the genus *Aliidiomarina* ubiquinone-9 was not detected [1].

Consequently, on the basis of the phylogenetic, physiological and chemotaxonomic data, strain IM 1326^T^ is considered to represent a novel species of the genus *Aliidiomarina *, for which the name *Aliidiomarina halalkaliphila* sp. nov. is proposed.

## Description of *Aliidiomarina halalkaliphila* sp. nov.

*Aliidiomarina halalkaliphila* (hal.al.ka.li’phi.la. Gr. masc. n. *hals halos* salt; N.L. n. *alkali* alkali; Gr. masc. adj. *philus* friend, loving; N.L. fem. adj. *halalkaliphila* salt and alkali-loving).

Cells are Gram-stain-negative, motile, rod-shaped, approximately 0.3–0.6 µm wide and 0.8–2.1 µm long. Colonies grown for 24 h at 37 °C on HM medium are circular, smooth, convex, light brown and approximately 1.0–3.0 mm in diameter. Oxidase and catalase are positive. Growth occurs at 4–45 °C (optimum, 37 °C), at pH 7.5–11.0 (optimum, pH 9.0–10.0) and with 0–13.0 % (w/v) NaCl concentration (optimum 4.0–6.0 %). Anaerobic and microaerobic growth does not occur on HM medium with or without nitrate. Nitrate is reduced, but nitrite is not. H_2_S is produced from cysteine. Production of indole and utilization of urea does not occur. The methyl red reaction is negative, but the Voges–Proskauer test result is positive. Gelatin, casein, DNA and Tweens 20, 40 and 60 are hydrolysed, but Tween 80, aesculin and starch are not hydrolysed. The following substrates are utilized as single carbon and energy sources for growth: l-arginine, l-lactate, l-glutamate, acetate, l-malate, pyruvate, succinate, citrate, l-serine, glycine, valine and histidine. The following substrates are not utilized as sole sources of carbon and energy: d-glucose, d-galactose, sucrose, d-sorbitol, fumarate, l-aspartate, mannose, maltose, starch, l-alanine, l-ornithine, d-fructose, l-sorbose, ribose, trehalose, d-xylose, glycerol and l-lysine. No acids are produced with the following substrates: d-glucose, d-galactose, sucrose, d-sorbitol, mannose, maltose, starch, d-fructose, l-sorbose, ribose, trehalose, d-xylose and glycerol. The respiratory quinone is ubiquinone-8. The major cellular fatty acids of the isolate are 10-methyl-C_16 : 0_/iso-C_17 : 1_ *ω*9*c* (summed feature 9), iso-C_15 : 0_ and iso-C_17 : 0_. The major polar lipids include diphosphatidylglycerol, phosphatidylglycerol, phosphatidylethanolamine and one unidentified aminophospholipid.

The type strain, IM 1326^T^ (=CGMCC 1.17056^T^=JCM 34227^T^), was isolated from the soda lake Hutong Qagan Lake in Inner Mongolia Autonomous Region, China. The genomic size of the type strain is 2.57 Mb, and DNA G+C content is 49.7 mol% (determined from the genome sequence).

## Supplementary Data

Supplementary material 1
