# Supplementary material for: Aliidiomarina halalkaliphila sp. nov., a haloalkaliphilic bacterium isolated from a soda lake in Inner Mongolia Autonomous Region, China
Source: Int J Syst Evol Microbiol. 2022 Mar 4;72(3):005263. doi: 10.1099/ijsem.0.005263 (PMC9558577; doi:10.1099/ijsem.0.005263)
Supplement: Supplementary material 1 [file ijsem-72-5263-s001.pdf]

Supplementary materials

***Aliidiomarina halalkaliphila* sp. nov., a haloalkaliphilic bacterium isolated from a soda lake in Inner Mongolia Autonomous Region, China**

Ming Yang[1,3], Qiong Xue[1,2], Zhenqiang Zuo[1,2], Jian Zhou[1], Shengjie Zhang[1,2], Ming Li[1], Heng Zhou[1], Manqi Zhang[1,2], Sumit Kumar[4], Wei Li[3], Guiying Chen[3], Dahe Zhao[1*], Hua Xiang[1,2*]

Author affiliations:

[1] State Key Laboratory of Microbial Resources, Institute of Microbiology, Chinese Academy of Sciences, 100101, Beijing, People's Republic of China

[2] University of Chinese Academy of Sciences, 100049, Beijing, People's Republic of China

[3] Sichuan Normal University, 610101, Sichuan, People's Republic of China

[4] Enzyme and Microbial Biochemistry Lab, Department of Chemistry, Indian Institute of Technology, Delhi, India

Corresponding authors:

Hua Xiang, xiangh@im.ac.cn; Dahe Zhao, zhaodh@im.ac.cn

Supplementary tables

Table S1 Comparison of general genome features about strain IM 1326^T and closely related species.

	Aliidiomarina halalkaliphila IM 1326^T	*Aliidiomarina taiwanensis* AIT1^T	*Aliidiomarina haloalkalitolerans* AK5^T	*Aliidiomarina sanyensis* GYP-1^T	*Aliidiomarina shirensis* AIS^T	*Aliidiomarina minuta* MLST1^T	*Aliidiomarina iranensis* GBPy7^T	*Aliidiomarina soli* Y4G10-17^T	*Aliidiomarina maris* CF12-14^T	*Aliidiomarina celeris* F3105^T	*Aliidiomarina sedimenti* GBSy1^T
Genomic size (Mb)	2.57	2.53	2.68	2.60	2.71	2.96	2.65	2.80	3.02	3.10	2.85
Sequencing depth	1189X	100X	100X	100X	100X	100X	100X	200X	100X	100X	100X
Scaffold number	12	29	29	48	15	7	36	54	44	11	10
Contig number	19	29	29	48	15	7	36	54	44	11	10
Contig N50 (bp)	337762	483587	251715	177650	735273	2140216	228613	705659	221091	558816	496752
GC content (mol %)	49.7	48.7	49.3	50.8	46.3	48.7	46.8	49.6	50.1	51.6	52.1
Protein count	2295	2300	2381	2329	2421	2751	2336	2557	2761	2860	2467
rRNA	3	3	12	3	10	5	9	12	11	10	8
tRNA	49	47	51	49	54	52	55	53	54	53	52
other RNA	4	4	4	4	4	4	4	4	4	4	4
Sequencing technology	Illumina NovaSeq	Illumina	Illumina	Illumina	Illumina	Illumina	Illumina	Illumina HiSeq	Illumina	Illumina	Illumina
Assembly method	SPAdes v. 3.13.0	SPAdes v. 3.7.0	SPAdes v. 3.7.0	SPAdes v. 3.7.0	SPAdes v. 3.7.0	SPAdes v. 3.7.0	SPAdes v. 3.7.0	ABySS v. 2.0.2	SPAdes v. 3.7.0	SPAdes v. 3.7.0	SPAdes v. 3.7.0

Table S2 Fatty acid content (%) of strain IM 1326^T and the type strain of related taxa. Strains: 1, IM 1326^T, this study; 2, *A. taiwanensis* CGMCC 1.15255^T; 3, *A. haloalkalitolerans* DSM 24939^T. Summed Feature 3: $C_{16:1}$ω7c/$C_{16:1}$ω6c; Summed Feature 7: unknown18.846/$C_{19:1}$ω6c/ cyclo-$C_{19:0}$; Summed Feature 8: $C_{18:1}$ω6c/$C_{18:1}$ω7c; Summed Feature 9: iso-$C_{17:1}$ω9c/10-methyl-$C_{16:0}$.

Fatty acids	1	2	3
$C_{12:0}$	1.0	0.7	0.49
$C_{14:0}$	2.1	1.5	1.46
$C_{16:0}$	7.8	13.2	4.72
$C_{17:0}$	0.9	0.8	1.18
$C_{18:0}$	1.3	3.7	1.03
Iso-$C_{13:0}$	3.0	0.7	3.73
Iso-$C_{15:0}$	16.1	13.7	30.28
Iso-$C_{17:0}$	13.1	10.8	15.12
Iso-$C_{11:0}$3-OH	5.5	3.5	7.57
Iso-$C_{15:0}$3-OH	3.3	0.3	5.54
Iso-$C_{15:1}$ F	4.0	2.3	4.29
Summed Feature 3*	6.0	7.3	2.71
Summed Feature 7*	0.7	0.1	0.03
Summed Feature 8*	5.0	9.6	1.46
Summed Feature 9*	22.2	17.5	13.93

*Summed Features are groups of two or three fatty acids that cannot be separated by GLC with the Microbial Identification System.

Supplementary figures

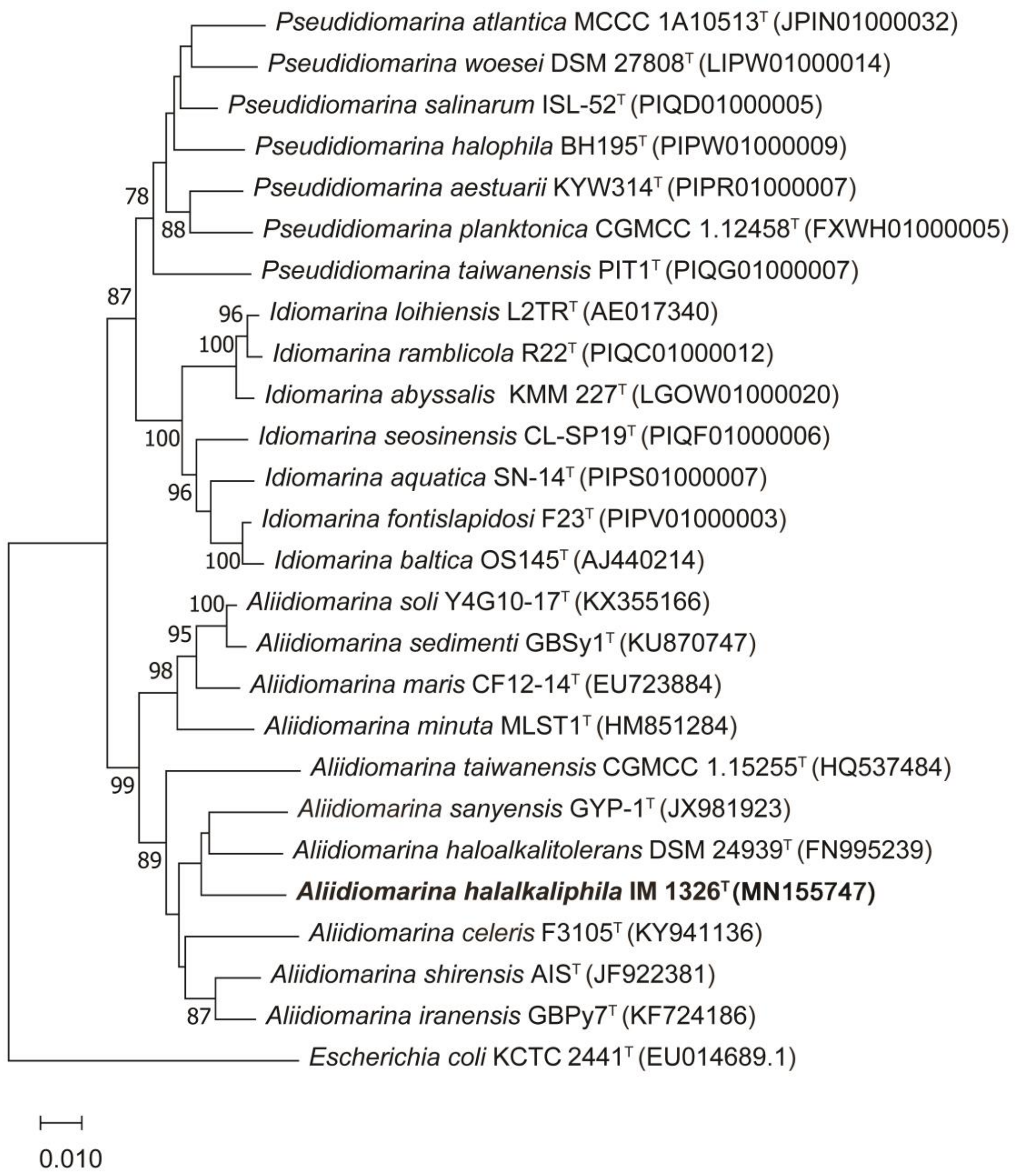

Fig. S1 Neighbor-joining phylogenetic tree based on 16S rRNA gene sequence showing the relationship between strain IM 1326^{T} and related taxa. Bootstrap values (%) were based on 1000 replicates and shown with more than 70% bootstrap support. The sequence of *Escherichia coli* KCTC 2441^{T} was used as outgroup. GenBank accession numbers are given in parentheses. Bar, 0.01 substitutions per nucleotide position.

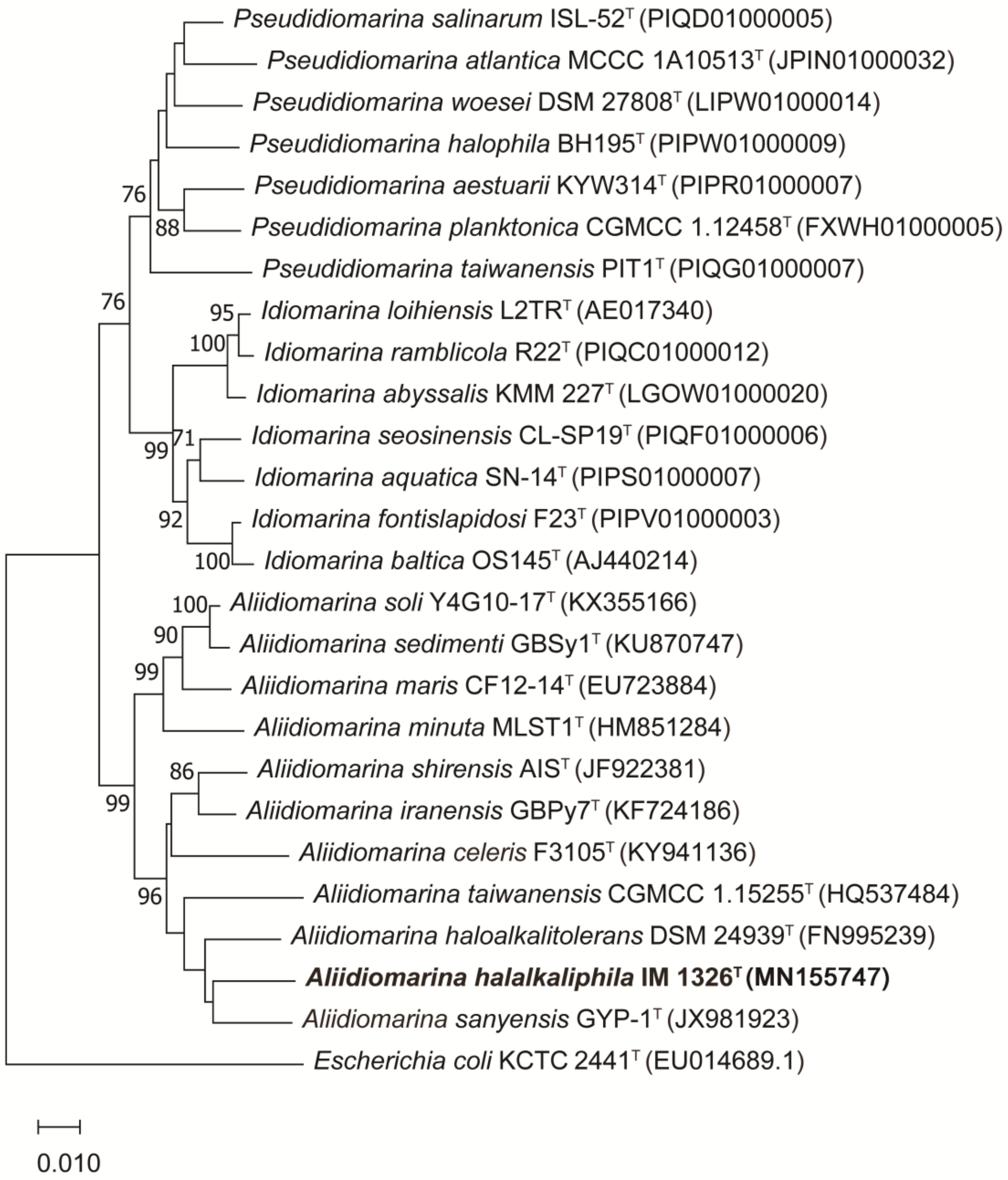

Fig. S2 Minimum Evolution phylogenetic tree based on 16S rRNA gene sequence showing the relationship between strain IM 1326^T and related taxa. Bootstrap values (%) were based on 1000 replicates and shown with more than 70% bootstrap support. The sequence of *Escherichia coli* KCTC 2441^T was used as outgroup. GenBank accession numbers are given in parentheses. Bar, 0.01 substitutions per nucleotide position.

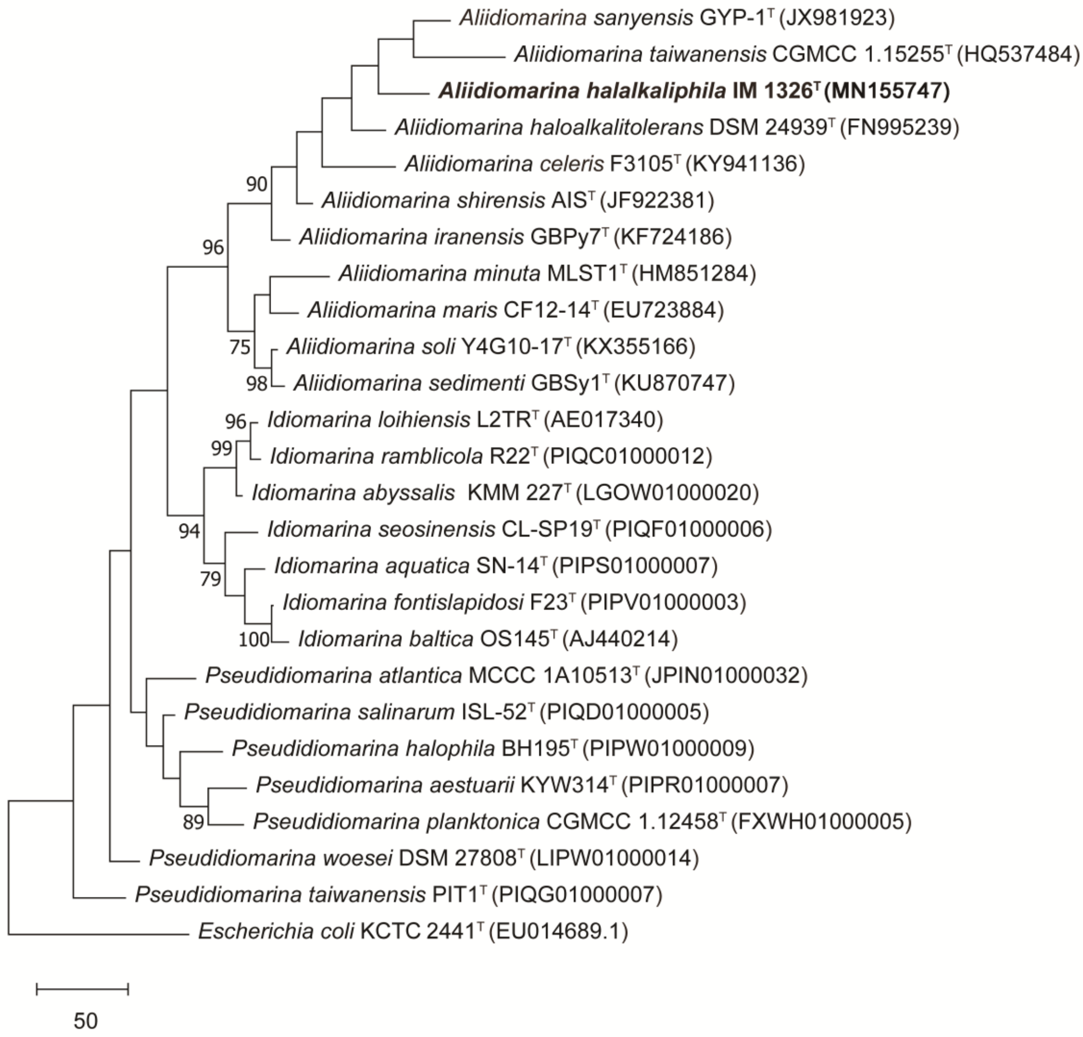

Fig. S3 Maximum-parsimony phylogenetic tree based on 16S rRNA gene sequence showing the relationship between strain IM 1326^{T} and related taxa. Bootstrap values (%) were based on 1000 replicates and shown with more than 70% bootstrap support. The sequence of *Escherichia coli* KCTC 2441^{T} was used as outgroup. GenBank accession numbers are given in parentheses.

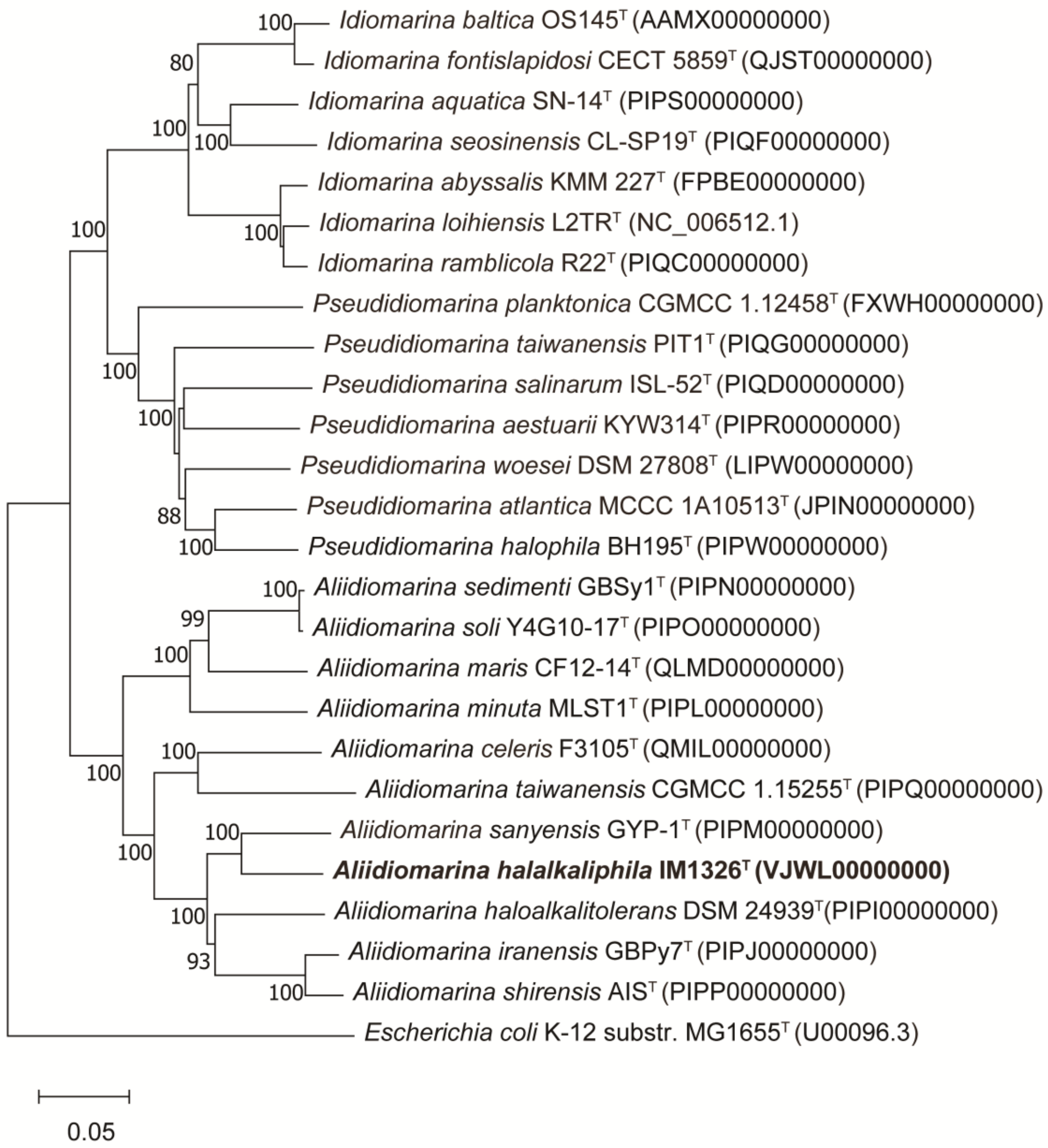

Fig. S4 Neighbor-joining phylogenetic tree based on bacterial 120 conserved single-copy genes sequence showing the relationship between strain IM 1326^T and related taxa. Bootstrap values (%) were based on 1000 replicates and shown with more than 70% bootstrap support. GenBank accession numbers were shown in parentheses. Bar, 0.05 substitutions per amino acid position.

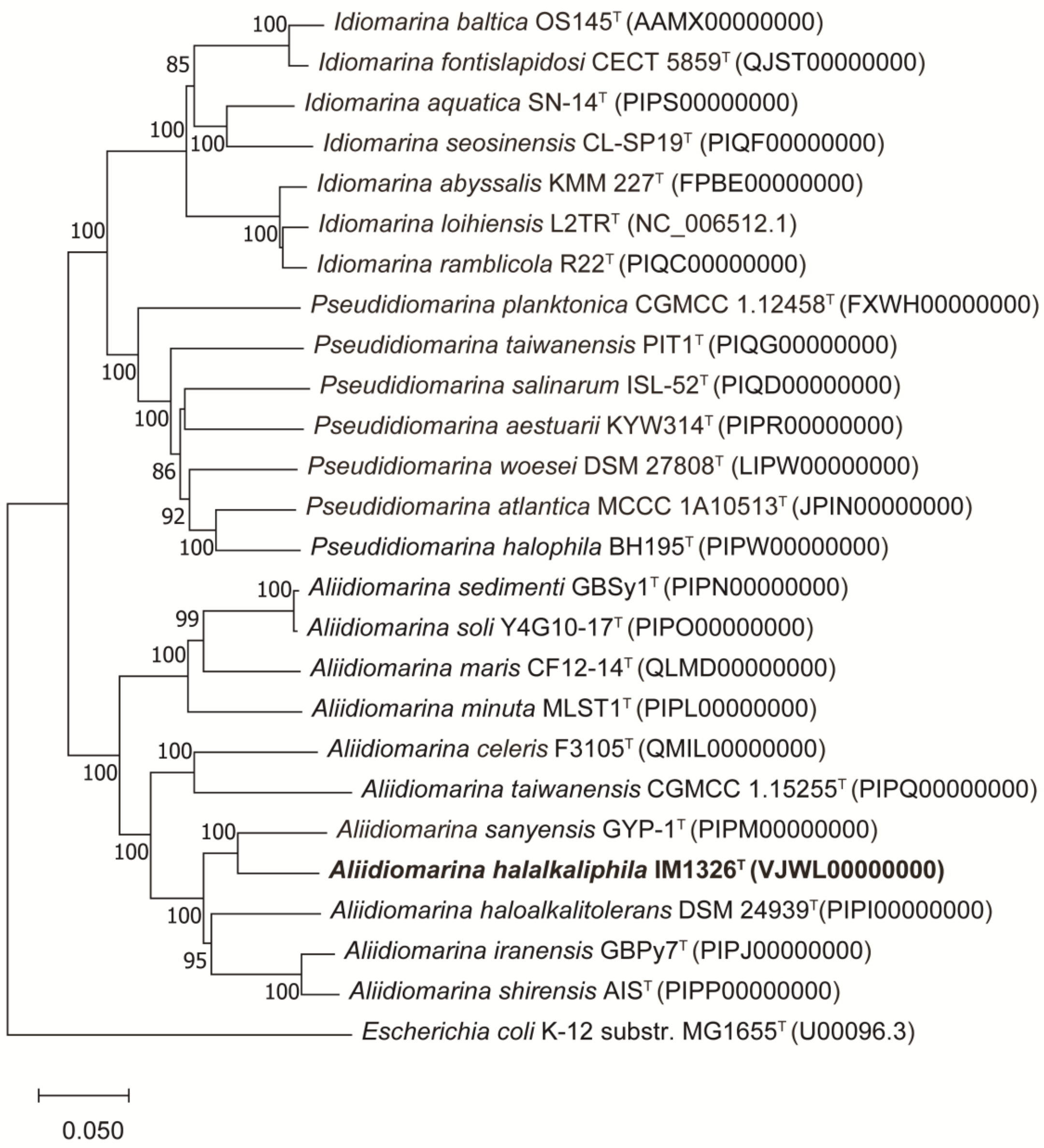

Fig. S5 Minimum Evolution tree based on bacterial 120 conserved single-copy genes sequence showing the relationship between strain IM 1326^T and related taxa. Bootstrap values (%) were based on 1000 replicates and shown with more than 70% bootstrap support. GenBank accession numbers were shown in parentheses. Bar, 0.05 substitutions per amino acid position.

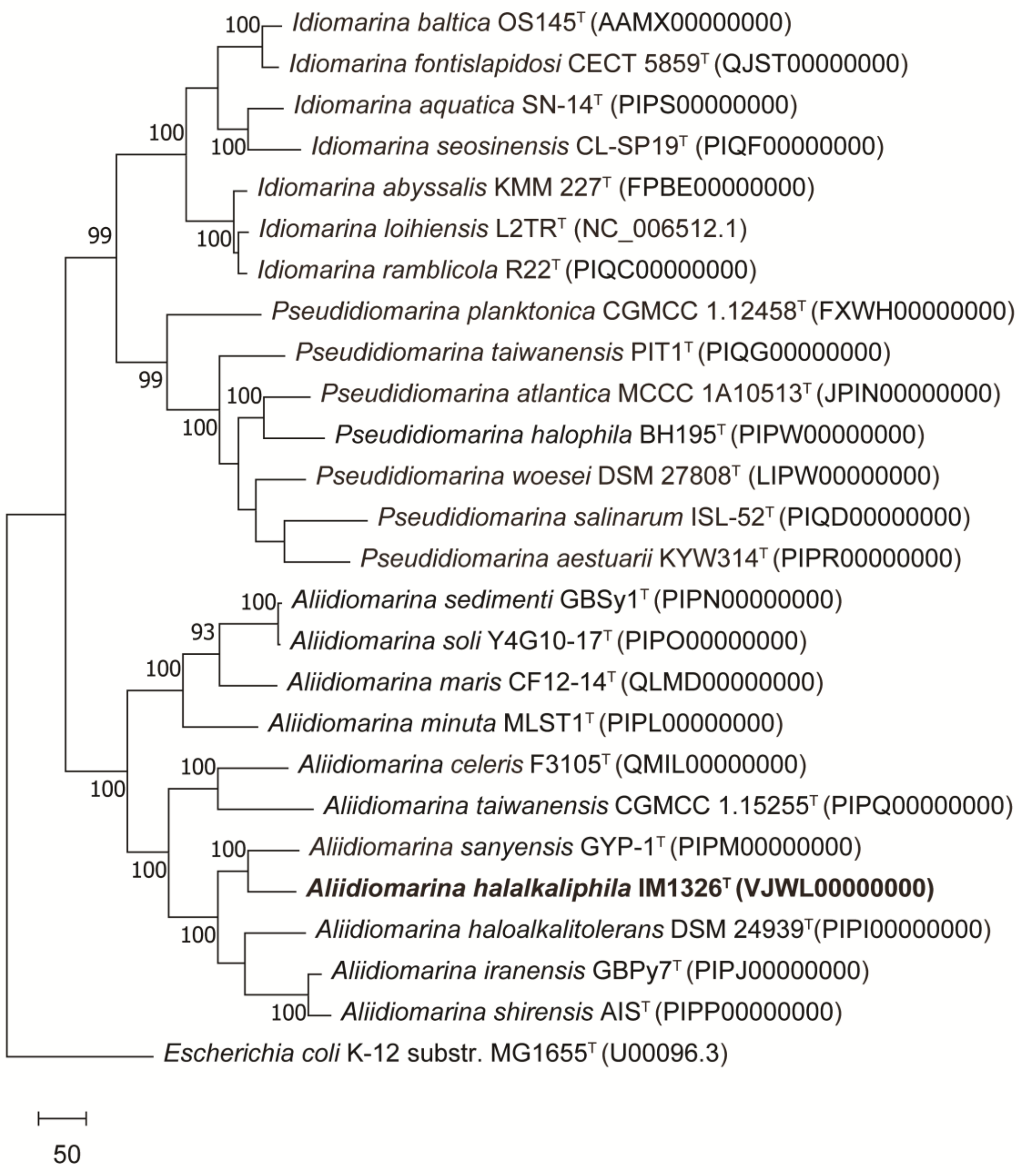

Fig. S6 Maximum-parsimony tree based on bacterial 120 conserved single-copy genes sequence showing the relationship between strain IM 1326^{T} and related taxa. Bootstrap values (%) were based on 1000 replicates and shown with more than 70% bootstrap support. GenBank accession numbers were shown in parentheses.

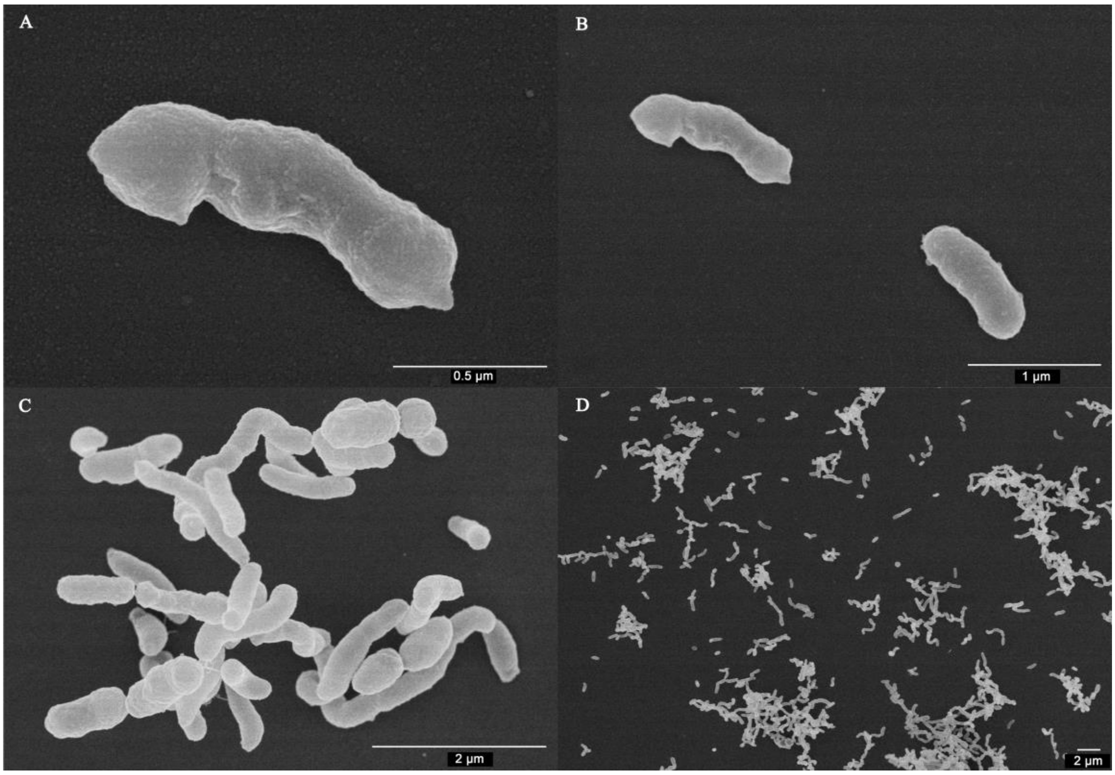

Fig. S7 Scanning electron microscopic image of strain IM 1326^T. Cells were grown on HM medium for 2 days at 37 °C. Bar: A, 0.5μm; B, 1 μm; C, 2 μm; D, 2 μm.

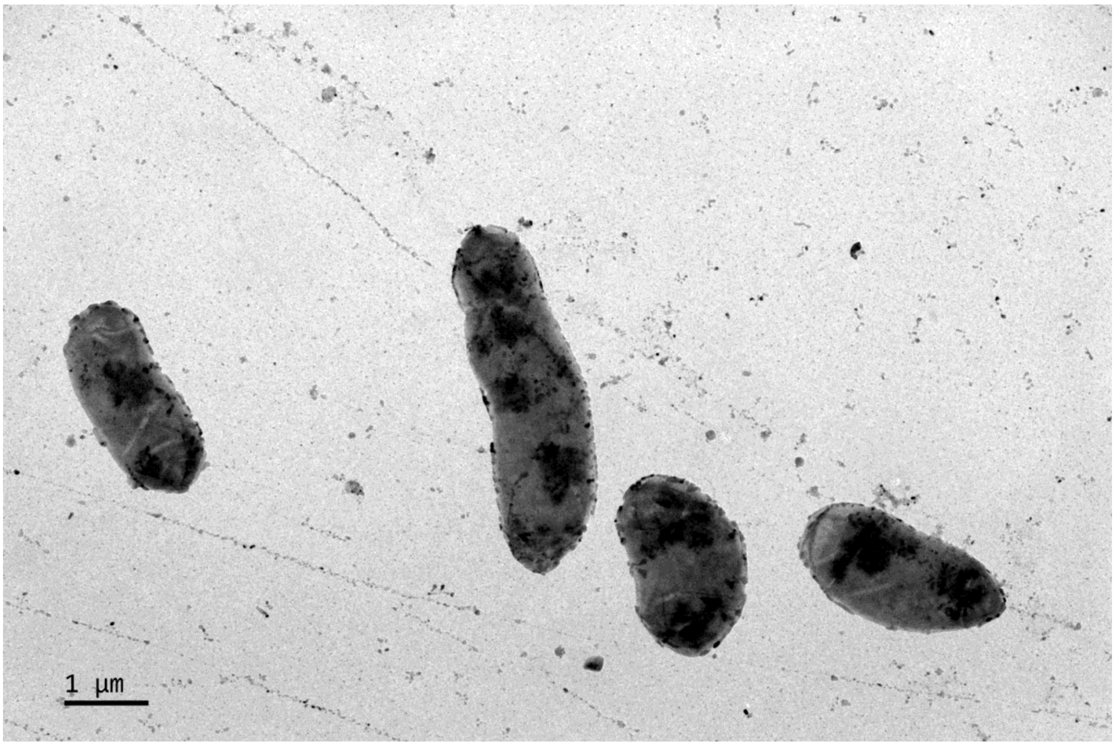

Fig. S8 Transmission electron microscopic image of strain IM 1326^T. Cells were grown on HM medium for 2 days at 37 °C. Bar: 1 μm.

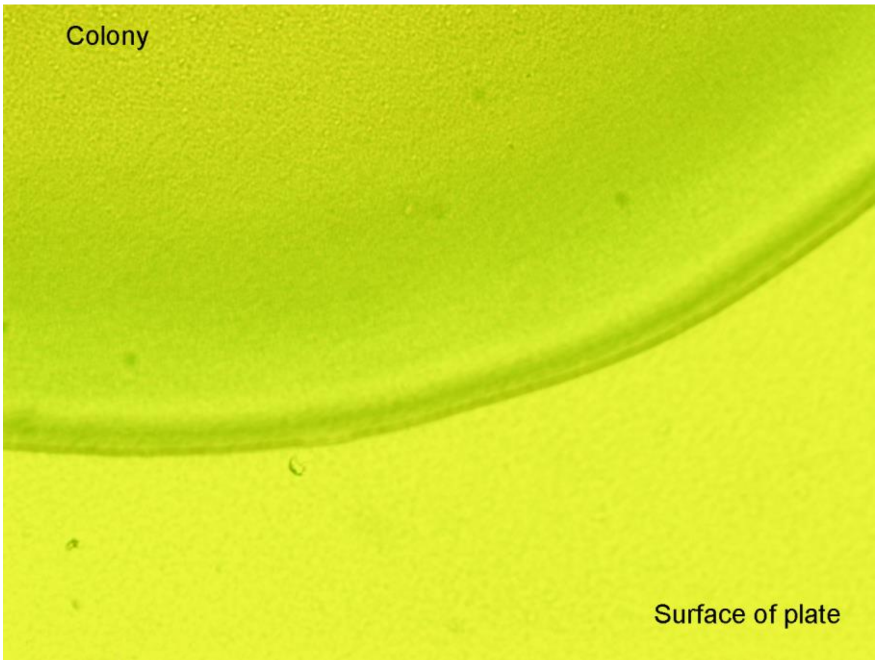

Fig. S9 Edge of the colony of the strain IM 1326^T on HM agar plate. Cells were grown for 2 days at 37 °C. The picture was the microscopic photograph using the normal optical microscope.

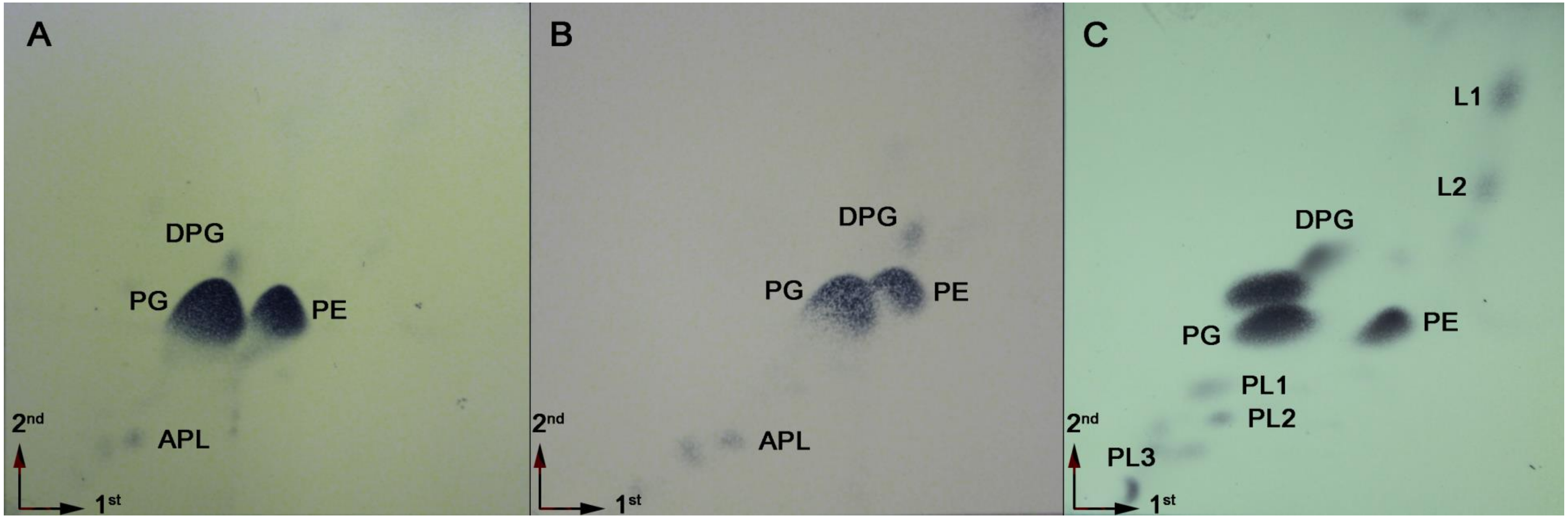

Fig. S10 Two-dimensional thin-layer chromatogram of polar lipids of *A. halalkaliphila* IM 1326^T (A), *A. taiwanensis* CGMCC 1.15255^T (B) and *A. halalkalitolerans* DSM24939^T (C). DPG, diphosphatidylglycerol; PG, phosphatidylglycerol; PE, phosphatidylethanolamine; APL, unidentified aminophospholipid; PL, phospholipid; L, lipid. PL1-3, unknown phospholipids; L1-2: unidentified polar lipids. 1st: first dimensional TLC, 2nd: second dimensional TLC.
